# Effects of Different Bud Thinning Methods on Nutritional Quality and Antioxidant Activities of Fruiting Bodies of *Pleurotus eryngii*

**DOI:** 10.3389/fpls.2022.917010

**Published:** 2022-06-16

**Authors:** Lingyun Liu, Lupeng Wang, Xuefei Li, Shurui Zhu, Niangang Pan, Xin Wang, Changtian Li, Yu Li

**Affiliations:** ^1^Engineering Research Center of Edible and Medicinal Fungi, Ministry of Education, Jilin Agricultural University, Changchun, China; ^2^International Joint Research Center, Creation of New Edible Mushroom Germplasm Resources, Ministry of Science and Technology, Jilin Agricultural University, Changchun, China

**Keywords:** *Pleurotus eryngii*, agronomic characters, nutritional components, antioxidant activities, buds

## Abstract

The cultivation of *Pleurotus eryngii* was studied by different methods, such as puncturing and fixed-point mushroom production, shading treatment at the top of the bag, and pulling the top of the bag. The agronomic characters, yield, nutritional components, and antioxidant activities *in vitro* of fruiting bodies of *P. eryngii* were determined. The results showed that the number of buds in the perforated treatment was less than that in the production method of traditional fruiting bodies to a certain extent. When a circular hole with a diameter of 1.5 cm was drilled in the perforated treatment, the number of buds was 5, which was less than that in the control group. The efficiency of artificial removal of buds was significantly higher than that of the control group, but the harvesting date was longer than that of other methods. The number of buds in shading treatment and bag opening treatment was significantly less than that in the control group, which could effectively control the number of buds and reduce the cost of manpower and material resources. In terms of nutritional components, the A3 treatment group with a hole diameter of 1.0 cm and a quantity of one had the highest crude protein content of 151.34 g, and a significant difference was observed in crude fiber content compared with other treatments. The extraction rate of B5 crude polysaccharide was the highest, and the extraction rate was 12.90%. The antioxidant activities *in vitro* increased with the increase of crude polysaccharide concentration. Using A3 treatment to cultivate fruiting bodies is conducive to meeting people’s requirements for improving quality of life.

## Introduction

*Pleurotus eryngii* is popular because of its rich nutrients and various active ingredients, including active polysaccharides, antibacterial peptides, and sterols (Yan et al., 2019). Compared with other edible fungi, *P. eryngii* has a higher content of protein, rich content of mannitol and free amino acids, and a lower content of fat and total sugar (Zhang et al., 2020). As the country with the largest production of edible fungi in the world, the annual output of *P. eryngii* was as high as 2.035 million tons in 2019, ranking seventh in the output of edible fungi (Mahari et al., 2020). In the Chinese market, *P. eryngii* mushrooms are generally big and thick, which is related to the character preference of some people in the 5,000 years of traditional Chinese culture. To cater to the mentality and preferences of consumers, producers shall sparse the buds and keep one or two buds to make them grow long and strong. The rapid industrial development of edible fungi also drives the development of *P. eryngii*, but in recent years, one of the reasons limiting the expansion of *P. eryngii* is still the problem of sparse buds (Papoutsis et al., 2020; Arunachalam et al., 2022). However, *P. eryngii* is different from *Flammulina filiformis*, *Pleurotus ostreatus*, *Hypsizygus marmoreus*, and other edible fungi. The problem of bud thinning is always dependent on artificial work. But with the rapid development of the economy, labor costs are becoming increasingly expensive. How to better sparse buds is an urgent problem to be solved in the industrial development of *P. eryngii*.

Zeng et al. (2014) used 20 cm × 20 cm kraft paper with a circular hole with a diameter of 4 cm in the middle to cover the mouth end of *P. eryngii*. The results showed that the buds could not differentiate in the area covered by kraft paper, so as to effectively control the buds of *P. eryngii*, in the cultivation process will produce more primordia, and the redundant buds need to be thinned manually. The number of sparse buds decreased from 2 to 1. *Pleurotus nebrodensis* is similar to *P. eryngii*. More mushroom buds will be produced in the process of culture, and the excess mushroom buds need to be removed manually. Zhang et al. (2017) showed that the process of scratching fungi on the material surface with one hole in the middle and scratching fungi on the material surface with two holes in the middle had an obvious effect on the formation of buds, with less formation of primordia and faster growth of fruiting bodies. Peng et al. (2016) studied the fixed-point selection of *P. nebrodensis*, and the results showed that “V-shaped” treatment reduced the area of primordia and the number of buds, saving labor and high biological efficiency.

The large and strong *P. eryngii* with a good appearance in China is not good in quality and taste. High-end markets will import *P. eryngii* from South Korea to meet people’s requirements on taste and quality. With the rapid development of the economy and the rapid improvement of people’s living standards, people’s requirements for quality are becoming more and more stringent nowadays (Petrescu et al., 2022; Truong et al., 2022). People’s requirements for the quality of life will no longer stay in the stage of full eating (Balkir et al., 2021; Toudert and Bringas-Rábago, 2021). At present, they are in the stage of good eating. In the future, they will tend to eat more nutritious (Shaheen et al., 2021; Liu et al., 2022). Due to this market demand and the high labor cost of traditional bud thinning, and after a series of measures, such as scratching fungi, strains with a single bud have not been found, indicating that varieties without bud thinning are extremely difficult to obtain. In this study, *P. eryngii* was treated by punching and fixed-point mushroom production, shading treatment at the mouth of the bag and pulling the mouth of the bag, and the agronomic characters, yield, nutritional components, and antioxidant activities of fruiting bodies of *P. eryngii* were determined, in order to provide a basis for the research on the mushroom production method of industrialized cultivation of *P. eryngii* and the replacement technology of manual bud thinning.

## Materials and Methods

### Material

Fungal strains: AT01 strain of *P. eryngii* was provided by the Engineering Research Center of Edible and Medicinal Fungi, Ministry of Education, Jilin Agricultural University.

### Methods

#### Experimental Design

Liquid culture medium: 25 g sucrose, 5 g KH_2_PO_4_, 2.5 g MgSO4, 2 g yeast extract powder, 1 L distilled water.

Culture materials: 38% sawdust, 30% corn cob, 20% wheat bran, 5% soy meal, 5% corn flour, 1% CaCO_3_, 1% lime, 65% moisture content, pH 6–7.

The experiment was conducted in the mushroom room of Jilin Yilong Changbaishan Industrial Co., Ltd. Antu County, China. *P. eryngii* was cultivated by wall type, and each row of the mushroom rack had 20 layers. The corner of polypropylene bag measuring 17 × 34 × 0.004 cm was used. The cover of the cultivation bag is provided with a filter. The weight of cultivation material was 1 kg and the inoculation amount was 100 g. The culture bags were sterilized for 2 h at 121°C with high temperature and high-pressure steam, and then, the liquid strains were added after cooling. The fungus chamber was cultured in secret until the mycelia ware full of bags, and then after ripening culture for 10 days, the mushroom was administered. The mycelia are cultured in the darkroom until the bag is full. After 10 days of post-ripening culture, it was managed in the humidified room.

#### Punch a Hole in a Fixed Position of a Bag

The mycelia full bag and pollution-free fungus package were selected, and the fixed-point mushroom test was conducted. Treatment A is a round hole and treatment B is a triangle hole. The size and number of holes are summarized in Table 1. For treatment A, round holes with different diameters and depths of about 1 cm were dug at the end of the cultivation bags with a knife. For treatment B, a knife was used to dig triangle holes with different side lengths and depths of about 1 cm at the end of the cultivation bags. The plastic was not cut off, and the holes were covered with the cut plastic bag film to prevent moisture evaporation. Each treatment group was set with 20 bags as repeats.

**TABLE 1:** Punch round holes and triangular holes in the fixed position of the bag.

Round hole	Diameters (cm)	Number	Triangular hole	Side length (cm)	Number
A1	0.5	1	B1	0.5	1
A2	0.5	2	B2	0.5	2
A3	1.0	1	B3	1.0	1
A4	1.0	2	B4	1.0	2
A5	1.5	1	B5	1.5	1
A6	1.5	2	B6	1.5	2

#### Shade the Top of the Bag

A total of 20 cultivation bags with the same growth were selected for shading treatment at the top of the bag. The mouth of the bag was wrapped with a black film and secured with a rubber band to prevent it from falling off. The place where the primordia were well developed was selected for opening, and hurting the primordia during the opening was avoided. This method is denoted as treatment C.

#### Bags Opening Treatment

A total of 20 cultivation bags with the same growth were selected for bag opening treatment. After opening the cover of the cultivation bag, the lower part of the bag mouth did not move, keeping the mycelia in contact with the bag and opening the upper part of the bag mouth. This method is recorded as treatment D.

#### Control Treatment

Conventional mushroom production management was adopted for the control treatment, and the operations, such as opening the cover, pulling the bag opening, taking the collar, manually bud thinning, and harvesting, were carried out in turn.

### Determination of Agronomic Characters

The agronomic characters of fruiting bodies of each treatment were counted, and the occurrence time of primordia, the number of mushrooms, and the characters of fruiting bodies were recorded. The weight of each treatment and yield of each bag were determined.

### Determination of Nutritional Components of Fruiting Bodies

According to the agronomic characters, the fruiting bodies of five treatments with better characters were selected for the determination of nutritional components. The content of total sugar was determined by the phenol-concentrated sulfuric acid method (Rover et al., 2013). Crude fat content was determined by the soxhlet extraction method (Nuchdang et al., 2022). The content of crude protein was determined by the Kjeldahl method (Zhao et al., 2022). The content of crude fiber was determined by the normal washing method (Wunjuntuk et al., 2022).

### Extraction of Crude Polysaccharide From Fruiting Bodies and Determination of Antioxidant Activities

According to the agronomic characters, the fruiting bodies of five treatments with better characters were selected for the determination of antioxidant activities. The fruiting bodies of *P. eryngii* in each group were thoroughly dried in a 45°C constant temperature oven, then crushed with a Chinese herbal medicine crusher, screened with 40 mesh, and kept in a 4°C refrigerator for standby. Refer to the method developed by Shu et al. (2019) to extract the crude polysaccharide of *P. eryngii*.

The total antioxidant capacity, hydroxyl radical, and superoxide anion radicals were measured with the corresponding kits of Nanjing Jiancheng Biotechnology Co., Ltd. Nanjing, China. The free radical scavenging ability was determined by the DPPH method (Mwangi et al., 2022). A total of 1, 2, 3, 4, and 5 mg/ml crude polysaccharides were used to determine the antioxidant activities *in vitro*.

### Statistical Analysis

The Origin 2018 (StatSoft Inc., Tulsa, Oregon, United States) software was used to draw a histogram. Agronomic characters were determined three times in each treatment, and 20 fruiting entities were determined in each treatment. For statistical analysis, we used SPSS statistical software package (SPSS Inc., Chicago, IL, United States). One-way analysis of variance (ANOVA) was performed. The Duncan test was used as the post-test of mean separation (*P* < 0.05).

## Results

### Effects of Different Treatments on the Emergence Time of Primordia and the Number of Buds of *Pleurotus eryngii*

It can be summarized from Table 2 that the primordia of A4, A5, A6, B5, and B6 treatments appeared 2 days earlier than the other treatments. In terms of production, shortening the time can improve efficiency, reduce costs, increase benefits, and promote the industrial development process of the *P. eryngii* industry. Through drilling, primordia appeared earlier, which may be due to the contact between mycelia and air after drilling, thus stimulating the formation of primordia. The traditional way to produce mushrooms is to open the cover of the bag so that the mycelia and the air had more contact. Due to the small drilling area, the number of primordia is less than that of the traditional way of mushroom extraction to a certain extent. The number of mushroom extraction will affect the number of buds. The number of buds in two holes is more than that in one hole, and the number of buds in A3 and A5 is less than the control group. The efficiency of perforated treatment was significantly higher than that of the control group, but the harvest date was 1 day later than that of C, D, and control treatments. The number of buds in treatment C and D was the least, which was significantly less than that in the control group, and there was a very significant difference with other treatments. It shows that A5, C, and D treatments can effectively control the number of buds and reduce the manpower and material resources consumed in thinning buds.

**TABLE 2:** Comparison of emergence time of primordia and number of buds under different treatments.

Treatment	Date of complete mycelia running	Date of primordia occurrence	Number of buds	Harvest date
A1	10.13	10.19	7.6 ± 2.40*bcde*	10.30
A2	10.13	10.19	9.8 ± 2.28*efg*	10.30
A3	10.13	10.19	5.4 ± 1.67*bcde*	10.30
A4	10.13	10.18	11.8 ± 2.38g	10.30
A5	10.13	10.18	5.4 ± 1.57b	10.30
A6	10.13	10.18	8.0 ± 1.41*bcde*	10.30
B1	10.13	10.19	8.4 ± 2.07*cde*	10.30
B2	10.13	10.19	8.4 ± 1.81*cde*	10.30
B3	10.13	10.19	6.4 ± 1.51*bcd*	10.30
B4	10.13	10.19	9.2 ± 2.58*def*	10.30
B5	10.13	10.18	6.8 ± 1.30*bcde*	10.30
B6	10.13	10.18	11.0 ± 1.58*fg*	10.30
C	10.13	10.20	3.2 ± 1.48a	10.29
D	10.13	10.20	3.0 ± 0.70a	10.29
CK	10.13	10.20	6.2 ± 1.48*bc*	10.29

*The data are mean ± standard deviation, and different letters in the same column indicate significant difference (P < 0.05).*

### Effects of Different Treatments on Agronomic Characters and Yield of Fruiting Bodies of *Pleurotus eryngii*

#### Effects of Different Treatments on Agronomic Characters of Fruiting Bodies

Different treatments had certain effects on the cap diameter, stipe diameter, and stipe length of fruiting bodies. It can be summarized in Table 3 that the longest cap diameter of treatment D is 65.71 ± 3.82 mm, which is significantly different from other treatments. In the fruiting bodies treated with perforation, only the cap diameter of the A3 treatment was larger than that of the control group. The stipe diameter of the D treatment was the longest, and there was a significant difference from other treatments except CK treatment. Considering the cap diameter, stipe diameter, and stipe length, the characteristics of D treatment and A3 treatment were better. Most of the perforated treatments had poor properties, and the cap diameter and stipe diameter were smaller than those of the control group. It may be that the fruiting body was in direct contact with the air after drilling, there was no cover, and the humidity was difficult to maintain, resulting in the buds. If the mushroom is produced by punching, the humidity of the mushroom room needs to be increased to a certain extent. In the treatment of circular hole drilling, when the hole diameter is the same, the more the number of holes, the smaller the cap diameter and stipe diameter of the fruiting bodies. The perforation diameter should not be too small or too large, so in all-round hole treatments, the cap diameter and stalk diameter of the A3 treatment are larger than those of other treatments. B treatments are triangular holes. Under the condition of the same side length, the number of holes has a less effect on the cap diameter and stipe diameter of fruiting bodies. The cap diameter and stipe diameter of fruiting bodies in the B5 treatment were larger than those in other treatments. Compared with the control treatment, the fruiting bodies of C treatment were larger, and the stipes were thin and long. Compared with the control treatment, the fruiting bodies of D treatment were larger, the stipes were thick and long, and the commodity character was good. Based on the above results, the agronomic characters of D treatment and A3 treatment were better than other treatments, and this method was more suitable for bud thinning of *P. eryngii*.

**TABLE 3:** Effects of different treatments on agronomic characters of fruiting bodies.

Treatment	Cap diameter (mm)	Stipe diameter (mm)	Stipe length (mm)
A1	51.17 ± 5.36*def*	41.17 ± 2.68*cd*	113.73 ± 3.82*bcde*
A2	46.63 ± 0.67*efg*	39.44 ± 1.25*cde*	123.53 ± 4.34b
A3	59.22 ± 5.57b	43.25 ± 5.50*bc*	113.33 ± 21.68*bc*
A4	47.54 ± 3.02*efg*	36.10 ± 3.31*def*	108.86 ± 13.32*cdef*
A5	50.47 ± 1.03*def*	40.17 ± 3.70*cde*	117.33 ± 7.04*bcd*
A6	49.84 ± 2.08*def*	39.46 ± 1.81*cde*	90.23 ± 4.93h
B1	42.67 ± 1.16g	33.12 ± 2.09f	104.16 ± 10.19*dfeg*
B2	48.01 ± 1.91*defg*	36.50 ± 3.12*def*	94.03 ± 10.12*gh*
B3	45.66 ± 5.11*fg*	34.52 ± 4.44*ef*	101.10 ± 3.68*fegh*
B4	45.26 ± 1.17*fg*	37.23 ± 1.64*cdef*	95.96 ± 4.79*fgh*
B5	51.86 ± 1.58*de*	40.64 ± 4.55*cde*	116.60 ± 6.35*bcd*
B6	49.51 ± 1.54*def*	40.66 ± 1.03*cde*	105.67 ± 2.89*defg*
C	57.64 ± 4.65*bc*	42.15 ± 2.40*cd*	127.23 ± 9.88b
D	65.71 ± 3.82a	51.17 ± 3.31a	139.60 ± 7.68a
CK	55.65 ± 2.54*cd*	47.97 ± 3.91*ab*	112.50 ± 6.22*bc*

*The data are mean ± standard deviation, and different letters in the same column indicate significant difference (P < 0.05).*

#### Effects of Different Treatments on Yield of Single Bag and Weight of Single Fruiting Body

**FIGURE 1:**
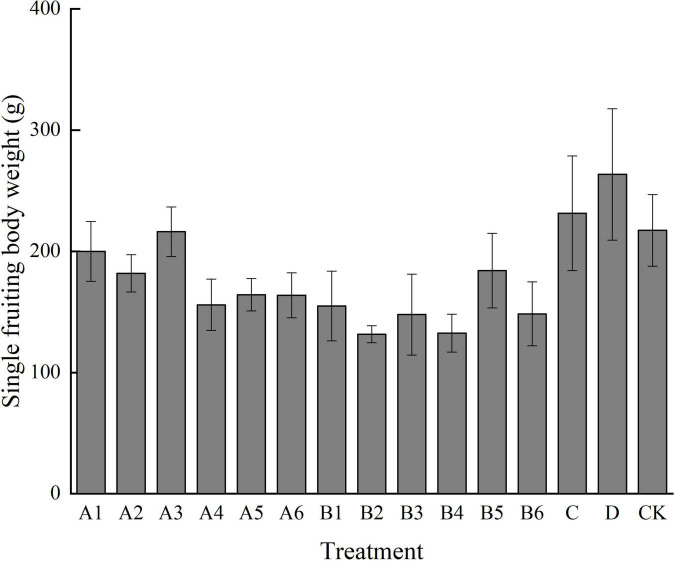
Effects of different treatments on the weight of single fruiting bodies. Different letters in the figure indicate significant differences (*P* < 0.05).

**FIGURE 2:**
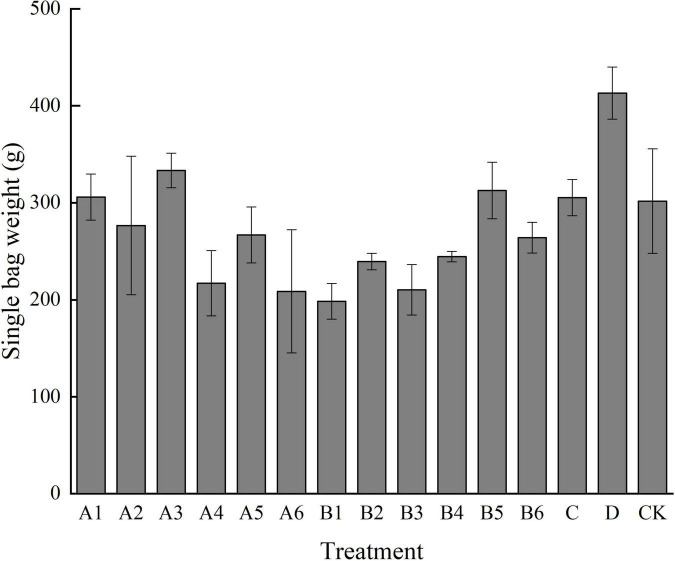
Effects of different treatments on the weight of single bag. Different letters in the figure indicate significant differences (*P* < 0.05).

It can be seen from Figures 1, 2 that different mushroom production methods have a certain impact on the yield and weight of *P. eryngii*. The single bag yield of A1, A3, B5, C, and D treatments was higher than that of the control group. The weight of the single bag in D treatment was 413.10 g, and that in the control treatment was 301.80 g. Among all treatments, only D treatment showed a significant difference from the control treatment. In terms of single mushroom weight, the maximum weight of D, C, CK, and A3 was 263.46 g, and the lowest weight was 216.16 g. Although there was a certain difference in weight, it did not reach a significant difference. In terms of treatment methods, under the same diameter (side length), the more are the number of holes, the smaller is the weight of a single mushroom and the lower is the yield. Therefore, when the drilling method is adopted, the number of circular holes and triangular holes should be one. In treatment A, the weight of the single bag and single mushroom of A3 is high. When drilling round holes, the suitable diameter is 1.0 cm, and the number of holes is one. In treatment B, the weight of the single bag and single mushroom of B5 was higher. When drilling triangular holes, the appropriate side length is 1.5 cm, and the number of holes is one. Bag opening treatment D had the highest single bag weight and single mushroom weight. Compared with traditional methods, all three methods can be adopted to save labor costs.

### Effects of Different Treatments on the Nutritional Quality of Fruiting Bodies of *Pleurotus eryngii*

According to the agronomic characters and yield, five treatments with better performance were selected as A3, B5, C, D, and control, and the crude fat, crude protein, crude fiber, total sugar, and ash content were used to determine the effects of different treatments on the nutritional components of fruiting bodies.

#### Effects of Different Treatments on the Crude Protein Content of Fruiting Body

**FIGURE 3:**
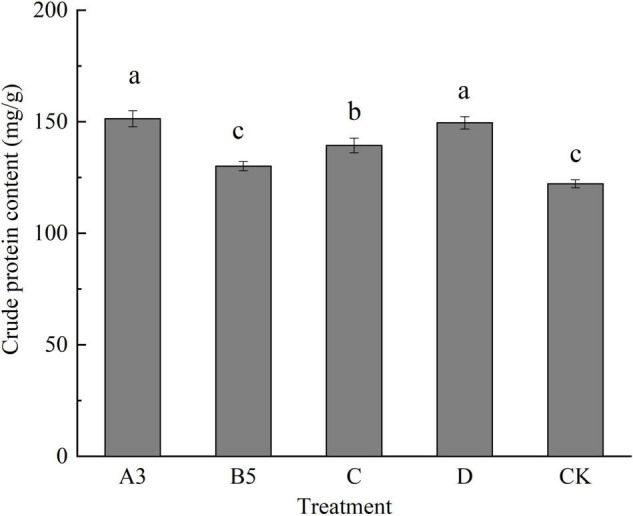
Crude protein content of fruiting bodies of each treatment. Different letters in the figure indicate significant differences (*P* < 0.05).

As shown in Figure 3, the crude protein content of fruiting bodies of each treatment ranged from 120.76 to 154.52 mg/g. In all the samples, the crude protein content was the highest in the A3 treatment group and the lowest in the control group, indicating that the crude protein content of fruiting bodies obtained by several different treatments was higher than that of the control group. Except for B5, the other treatments were significantly different from the control. It is possible that fewer treated primordia are present, and nutrients are retained for the growth and accumulation of other normal fruiting bodies. After treating, the fruiting bodies have high protein content and higher nutrient composition.

#### Effects of Different Treatments on the Crude Fiber Content of Fruiting Body

**FIGURE 4:**
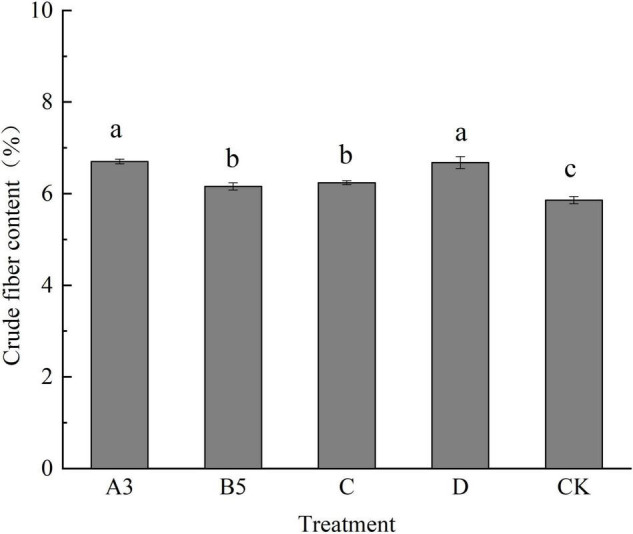
Crude fiber content of fruiting bodies of each treatment. Different letters in the figure indicate significant differences (*P* < 0.05).

As can be seen from Figure 4, the crude fiber content in fruiting bodies of each treatment ranged from 5.76 to 6.82%. The crude fiber content of A3 and D treatments was significantly different from that of other treatments, and the other four treatments showed significant differences compared with the control. The results showed that different treatments had certain effects on the crude fiber content of fruiting bodies.

#### Effects of Different Treatments on the Crude Fat Content of Fruiting Body

**FIGURE 5:**
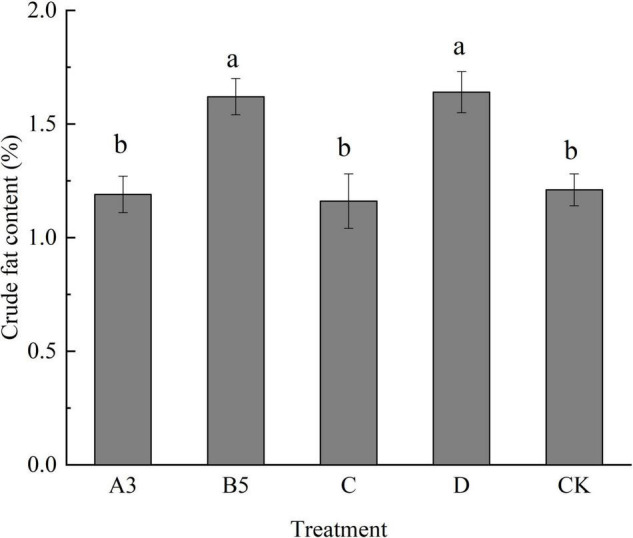
Crude fat content of fruiting bodies of each treatment. Different letters in the figure indicate significant differences (*P* < 0.05).

It can be seen from Figure 5 that the crude fat content of each treatment fruiting body was lower than that of the crude fiber. The crude fat content of B5 and D treatments was significantly different from that of other treatments. There was no significant difference between A3 and C treatment and control treatment. Different treatment methods have a certain impact on the crude fat content of fruiting bodies. The treatment of A3 and C is conducive to meeting people’s desire for a low-fat and healthy life.

#### Effects of Different Treatments on the Total Sugar Content of Fruiting Body

**FIGURE 6:**
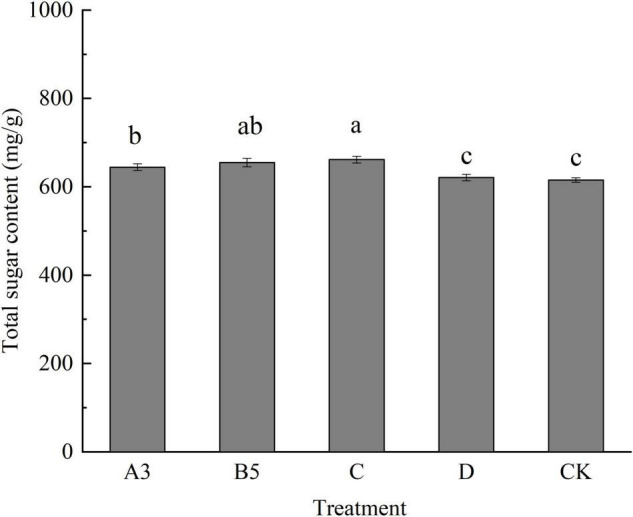
Total sugar content of fruiting bodies of each treatment. Different letters in the figure indicate significant differences (*P* < 0.05).

It can be seen from Figure 6 that there was a less difference in the total sugar content of each treatment for fruiting bodies. D and CK treatments were significantly lower than other treatments. The total sugar content of the B5 and C treatment was higher than that of other treatments. Different treatments had a certain effect on the total sugar content of fruiting bodies. B3 and C treatments were of great significance for supplementing human polysaccharides.

### Effects of Different Treatments on Antioxidant Activities of Crude Polysaccharide From Fruiting Bodies of *Pleurotus eryngii*

#### Effects of Different Treatments on Extraction Rate of Crude Polysaccharide From Fruiting Bodies of *Pleurotus eryngii*

According to agronomic characters and yield, five treatments with good performance were selected as A3, B5, C, D, and control. The polysaccharide of fruiting body was extracted to obtain the percentage of crude polysaccharide. It can be seen from Table 4 that the extraction rate of crude polysaccharide from the fruiting bodies of B5 treatment was the highest, which was 12.90%. The extraction rate of crude polysaccharide from fruiting bodies treated with CK was the lowest, which was 9.06%. Different treatment methods had certain effects on the extraction rate of crude polysaccharide from fruiting bodies. B5 with the highest extraction rate of crude polysaccharide was selected to determine the antioxidant activities *in vitro*.

**TABLE 4:** Extraction rate of crude polysaccharide from fruiting bodies of different treatments.

Treatment	A3	B5	C	D	CK
Crude polysaccharide(%)	9.20	12.90	12.23	10.78	9.06

#### Effects of Different Concentrations of Crude Polysaccharides From *Pleurotus eryngii* Fruiting Bodies on Total Antioxidant Capacity

**FIGURE 7:**
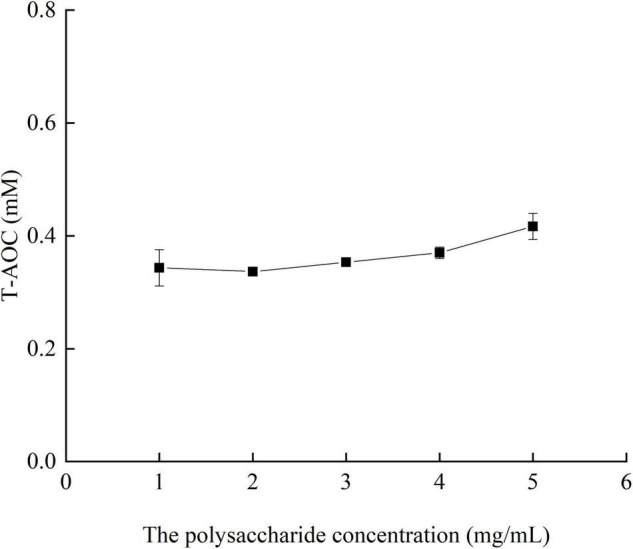
Total antioxidant capacity of crude polysaccharides from fruiting bodies at different concentrations *in vitro.*

It can be seen from Figure 7 that with the increase of the concentration of crude polysaccharide, the total antioxidant capacity *in vitro* also gradually increased. When the concentration of polysaccharide reached 5 mg/ml, its total antioxidant capacity was the strongest, reaching 0.41 mM.

#### Effects of Different Concentrations of Crude Polysaccharides From *Pleurotus eryngii* Fruiting Bodies on DPPH Radical Scavenging Ability

**FIGURE 8:**
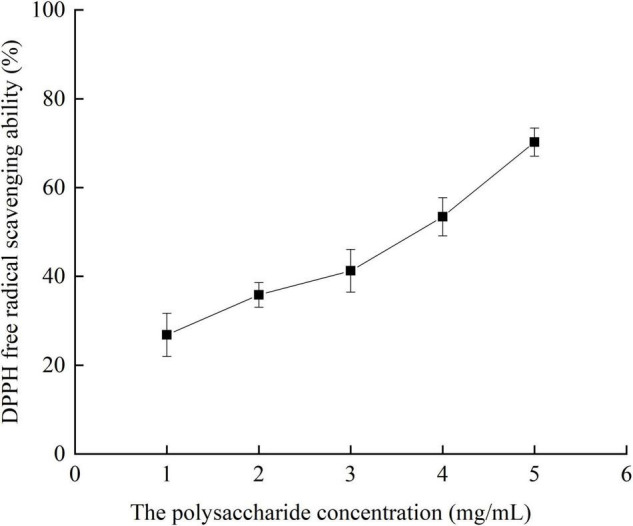
DPPH radical scavenging ability of crude polysaccharides from fruiting bodies with different concentrations *in vitro.*

It can be seen from Figure 8 that the scavenging capacity of DPPH radical increases with the increase of extract concentration. When the concentration is 5 mg/ml, the scavenging rate is the highest, reaching 70.23%.

#### Effects of Different Concentrations of Crude Polysaccharides From *Pleurotus eryngii* Fruiting Bodies on Hydroxyl Radical Scavenging Ability

**FIGURE 9:**
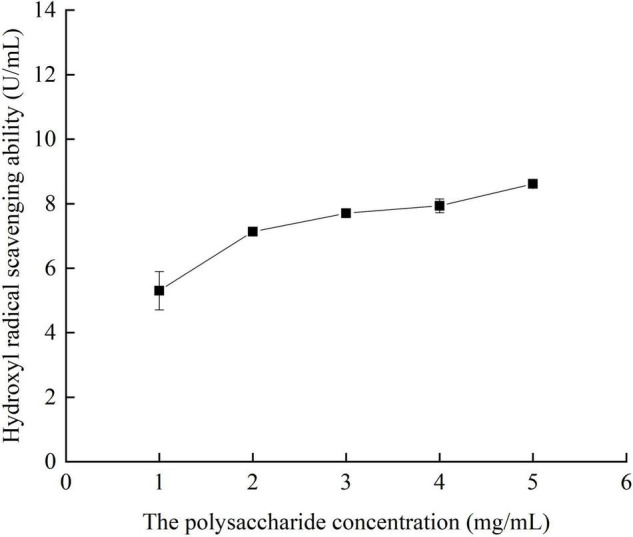
Hydroxyl radical scavenging ability of crude polysaccharides from fruiting bodies with different concentrations *in vitro.*

It can be seen from Figure 9 that when the concentration of polysaccharide was in the range of 1.0–5.0 mg/ml, the hydroxyl radical scavenging ability increases with the increase of polysaccharide concentration, up to 9 U/ml.

#### Effects of Different Concentrations of Crude Polysaccharides From *Pleurotus eryngii* Fruiting Bodies on Superoxide Anion Scavenging Ability

**FIGURE 10:**
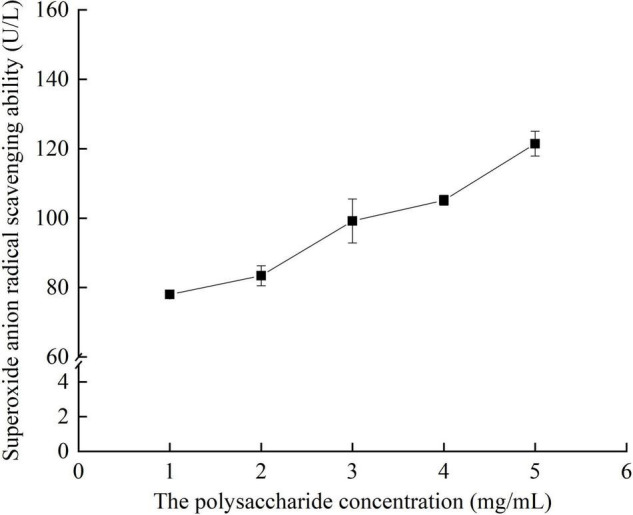
Superoxide anion radical scavenging ability of crude polysaccharides from fruiting bodies with different concentrations *in vitro.*

It can be seen from Figure 10 that the crude polysaccharide extract of *P. eryngii* fruiting body had the ability to scavenge superoxide anion free radical. The scavenging capacity increased with the increase of extract concentration. When the concentration reached 5 mg/ml, the scavenging capacity was the strongest, up to 121.46 U/L.

## Discussion

The emergence time of primordia and the number of buds were affected by different methods. This result may be used to stimulate the formation of primordia by contacting the mycelia with the air after drilling. Traditionally, the mycelia will have more contact with the air after opening the cover.

The agronomic characteristics of fruiting bodies are closely related to the size of perforation; a too small perforation can inhibit the lateral growth of fungus stalks, and a too large perforation can easily form multiple buds. Therefore, an appropriate opening size cannot only promote the lateral growth of the stipes but also avoid the workload of artificial bud thinning caused by excessive buds. Appropriate opening size can also promote the yield and the weight of a single mushroom, so the selection of an appropriate treatment method cannot only solve the problem of high labor cost in production but also have a significant promotion effect on the yield.

Edible fungi are a good source of dietary protein, fiber, fat, carbohydrates, vitamins, and minerals (Chien et al., 2015; Maity et al., 2021). *P. eryngii* is rich in functional fiber and has antioxidant and immune-stimulating properties (Li and Shah, 2016). Kleftaki et al. (2022) showed that *P. eryngii* could improve postprandial blood glucose, hunger, and satiety, and enhance auxin-releasing peptide inhibition and satiety, which could be attributed to bioactive polysaccharides. In recent decades, extensive studies have shown that mushroom polysaccharides have a variety of health-promoting effects, namely, anti-tumor, anti-oxidation, anti-inflammation, anti-obesity, anti-diabetes, and regulation of lipid absorption and metabolism (Yin et al., 2020; Aamri et al., 2022).

Crude protein is a general term for protein and nitrogen-containing compounds. Edible fungi contain more protein, with the protein content of fruit bodies accounting for 3–4% of fresh weight or 10–40% of dry weight, between meat and vegetables, and 3–6 times of common vegetables (Oliveira do Carmo et al., 2021). Different treatments had certain effects on the crude protein content of fruiting bodies, and the crude protein content of all treatments except B5 was significantly different from that of the control. This indicates that in the process of innovation, not only high yield can be obtained, but also the nutrient composition of the cultivation material can be retained greatly, to provide the fruiting bodies with fewer primordia to grow.

The main component of crude fiber is cellulose, but there are still some other components, such as hemicellulose, pentosan, and a small amount of nitrogen-containing non-protein impurities, so it is called crude fiber (Keerthana et al., 2020). *P. eryngii* is rich in dietary fiber, which can be divided into soluble and insoluble dietary fibers. Insoluble dietary fiber, namely crude fiber, can regulate the blood sugar content in the human body and treat diabetes (Teniou et al., 2022). Dietary fiber can also be combined with cholic acid, to reduce cholesterol levels, prevention, and treatment of coronary heart disease. At the same time, it can absorb ions and maintain the balance of sodium and potassium ions in the body, thus reducing blood pressure (Chang et al., 2021). With the rapid development of the economy, the material level of people is improving rapidly, and more and more diseases are affecting the younger population (Fernandes et al., 2022). More and more young people have a variety of diseases, such as obesity, high blood sugar, high blood fat, and high cholesterol caused, by excess nutrition (Lin et al., 2013). To prevent such diseases, people now pay more attention to foods with high fiber and low fat. In daily life, people will avoid related diseases by properly eating foods rich in crude fiber (Pathania and Kaur, 2022). The crude fiber content of each treatment was significantly higher than that of the control treatment, which also indicated that the fruiting bodies after treatment were very suitable for contemporary people. When eating *P. eryngii*, it could not only supplement protein but also supplement more crude fiber.

Obesity is a serious health problem, and its severity is spreading. Globally, 13% of adults were obese and 39% were overweight in 2014, according to the WHO (Shemirani et al., 2021). Obesity is associated with multiple diseases, such as type 2 diabetes mellitus (T2DM), cardiovascular disease, neurological diseases, and cancer, increasing morbidity and mortality (Lim et al., 2012). To better prevent obesity, the intake of a low-calorie, low-fat, and low-carbohydrate diet has a significant effect on weight loss and fat content (Christensen et al., 2021). In this context, the lower crude fat content of *P. eryngii* is more suitable for the market demand, so A3 and C treatments better meet the market demand.

Total sugars include monosaccharides and polysaccharides. Several linear/branching glucans isolated from edible fungi have a wide range of biological activities, such as antibacterial, antihypertensive, antifungal, anti-inflammatory, antiviral, antibacterial, hepatoprotective, antidiabetic, low-fat, antitumor, immune, antithrombotic, and antioxidant (Manna et al., 2017; de Jesus et al., 2018). Most carbohydrates occur in nature in the form of polysaccharides. Polysaccharides are relatively complex carbohydrates and are considered to be the first biopolymers to form on Earth (Tolstoguzov, 2004). A variety of polysaccharides from different natural sources, such as mushrooms (Meng et al., 2016; Yin et al., 2020) and plants (Panda et al., 2015), are being recognized as a supplement for increased health benefits. The supplementation of total sugar in *P. eryngii* is an excellent source of polysaccharide intake, and treatment with B3 and C plays an important role in increasing polysaccharide content.

The polysaccharides, polyphenols, and other substances contained in the fruiting body of *P. eryngii* have antioxidant activities (Wang et al., 2014). The total antioxidant capacity showed that polysaccharide was an effective electron donor, which could react with reactive oxygen species and convert it into more stable products (Wei et al., 2018). The antioxidant capacity *in vitro* is usually evaluated by DPPH radical, hydroxyl radical, superoxide anion radical scavenging capacity, and total antioxidant capacity (Wang et al., 2020). DPPH assay is one of the most widely used assays to evaluate the free radical scavenging ability of compounds or the antioxidant activity of extracts (Oktay et al., 2015). The elimination of hydroxyl radicals may be one of the most effective methods to prevent oxidative damage of cells *in vivo* (Yang et al., 2014). Superoxide anion radicals have longer reaction time than other radicals, and they can further participate in the reactions leading to the formation of more reactive oxygen species (Su and Li, 2020). In this study, the total antioxidant capacity, DPPH radical, hydroxyl radical, and superoxide anion radical scavenging capacity increased with the increase of crude polysaccharide concentration in the fruiting body.

In this context, it is very important to treat *P. eryngii* in different ways. It cannot only solve the problem of bud thinning but also improve the yield and quality of *P. eryngii*. People’s requirements for the high added value of modern food are met. Saving cost, increasing output, and increasing the added value of products lay a good theoretical and practical foundation for the industrial development of *P. eryngii*. Mushrooms filled with numerous bioactive nutraceuticals can be used as an active ingredient for the preparation of functional foods, which may promote health by improving immune strength, prevention and/or reduction of cancer risks, protection of the nervous system damage, etc. (Su and Li, 2020). Vamanu and Gatea (2020) have shown that data on the role of human microbiota in maintaining overall health are increasingly used by the scientific community. Edible fungi have an obvious anticancer effect, and the bioactive molecules contained in edible fungi show low toxicity and have good tolerance in the human body (Kornienko et al., 2015). Until now, 80–85% of bioactive compounds that mushrooms contain are extracted from the fruiting bodies, and they belong to many chemical groups, such as polysaccharides, polyketides, phenolic compounds, triterpenoids, steroids, proteins, nucleotides, fatty acids, and lactones (Elisashvili, 2012). The polysaccharide of *Pleurotus eryngii* also has medicinal properties, such as antioxidant and antitumor activities (Jing et al., 2013). The results showed that adding 5% mushroom mycelium to bread instead of wheat would not affect its rheology, texture, and sensory factors, and there were still high concentrations of ergotathione and aminobutyric acid after baking (Ulziijargal et al., 2013). It has developed functional compounds to promote the biological activity of human microbiota and regulate human health. These compounds can be used as nutritional and health products, drugs, or food supplements, so as to produce more valuable metabolic pathways of bioactive compounds (Lu et al., 2020). In the future, further research should be carried out on the correlation between the bioactivity of microbiota and the bioavailability of functional compounds (Divya and Pradeep, 2021).

## Conclusion

Through a series of studies, the results showed that bag opening treatment D could effectively control the number of buds and had a better performance of fruiting body’s agronomic traits, and the yield and weight of a single bag were the highest, which could reduce the manpower and material resources of bud thinning. In terms of nutritional components, the A3 treatment group with a hole diameter of 1.0 cm has the highest crude protein content and a significant difference in crude fiber content compared with other treatments. The extraction rate of B5 crude polysaccharide was the highest, with an extraction rate of 12.90%. The total antioxidant capacity *in vitro*, DPPH radical, hydroxyl radical, and superoxide anion radical scavenging capacity of crude polysaccharide from fruiting body increased with the increase of polysaccharide concentration. Using A3 treatment to cultivate fruiting bodies is conducive to meeting people’s requirements for quality of life.

## Data Availability Statement

The original contributions presented in this study are included in the article/Supplementary Material, further inquiries can be directed to the corresponding authors.

## Author Contributions

LL contributed to the methodology and writing and editing the original draft. LW contributed to the data curation, visualization, and investigation. XL contributed to the software and validation. SZ contributed to the software. NP and XW contributed to the validation. CL contributed to the conceptualization, methodology, writing and reviewing the manuscript. YL contributed to the conceptualization and methodology. All authors contributed to the article and approved the submitted version.

## Conflict of Interest

The authors declare that the research was conducted in the absence of any commercial or financial relationships that could be construed as a potential conflict of interest.

## Publisher’s Note

All claims expressed in this article are solely those of the authors and do not necessarily represent those of their affiliated organizations, or those of the publisher, the editors and the reviewers. Any product that may be evaluated in this article, or claim that may be made by its manufacturer, is not guaranteed or endorsed by the publisher.
