# Supplementary material for: Effects of Different Bud Thinning Methods on Nutritional Quality and Antioxidant Activities of Fruiting Bodies of Pleurotus eryngii
Source: Front Plant Sci. 2022 Jun 16;13:917010. doi: 10.3389/fpls.2022.917010 (PMC9244624; doi:10.3389/fpls.2022.917010)
Supplement: Supplementary file 2 [file Data_Sheet_1.docx]

**Supplementary Figure**


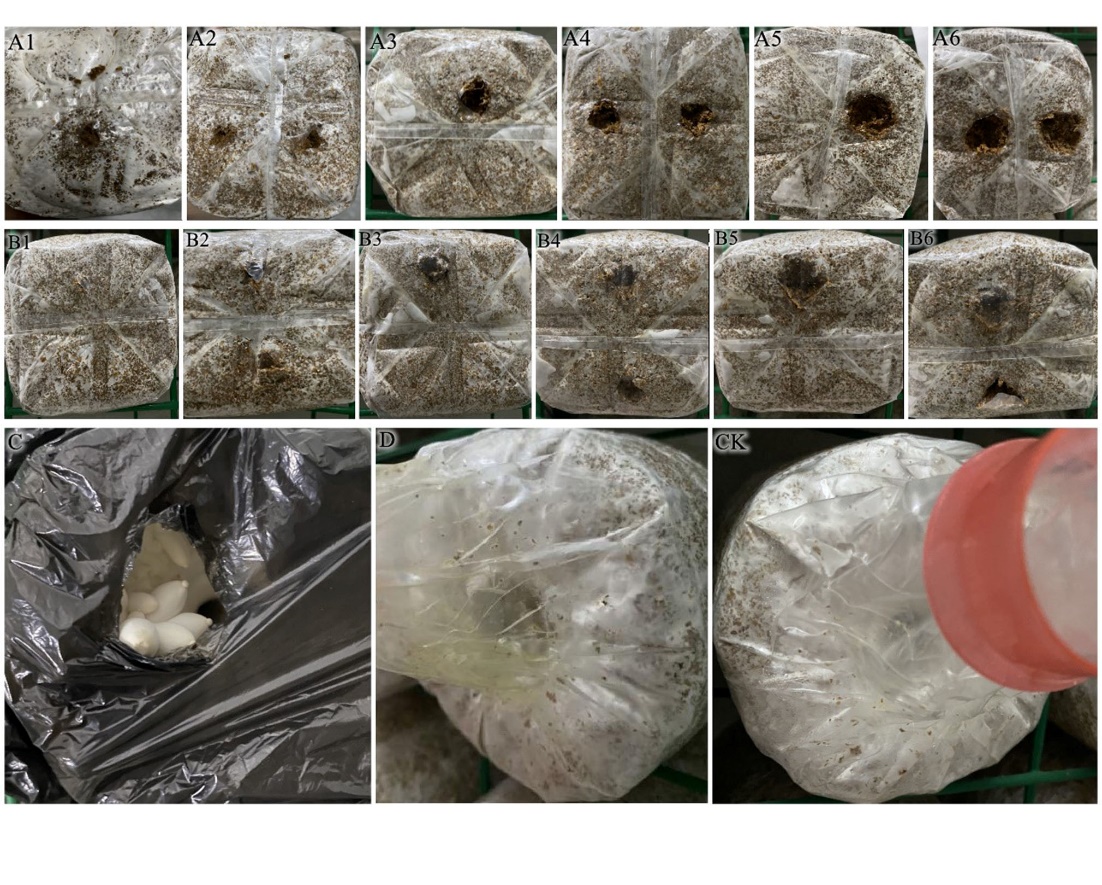


**Supplementary Fig. 1 Effects of different treatments on the number of fruiting bodies of *P. eryngii***
